# Development and validation of an instrument (CSII – Brazil) to assess users’ conceptual and procedural knowledge of continuous subcutaneous infusion systems

**DOI:** 10.20945/2359-3997000000314

**Published:** 2020-12-15

**Authors:** Camilla Magalhães de Oliveira Amaral, Carla Ferraz de Oliveira Borges, Adriana Silvina Pagano, Aleida Nazareth Soares, Gabriela Franco Mourão, Janice Sepúlveda Reis

**Affiliations:** 1 Ensino e Pesquisa – Santa Casa de Belo Horizonte Belo Horizonte MG Brasil Ensino e Pesquisa – Santa Casa de Belo Horizonte, Belo Horizonte, MG, Brasil; 2 Universidade Federal de Minas Gerais Faculdade de Letras Belo Horizonte MG Brasil Faculdade de Letras, Universidade Federal de Minas Gerais, Belo Horizonte, MG, Brasil

**Keywords:** Diabetes mellitus, insulin infusion system, knowledge, cultural adaptation, validation studies

## Abstract

**Objective:**

To develop, adapt and validate an instrument named “CSII – Brazil” to assess users’ conceptual and procedural knowledge of continuous subcutaneous insulin infusion systems.

**Materials and methods:**

Methodological and exploratory study developed in three stages: a) instrument development; b) content validation and cultural adaptation (evaluation by a committee of experts and pre-test with CSII users); c) psychometric validation through instrument application in a sample of 60 patients by means of the web tool e-Surv. Internal consistency and reproducibility analyses were performed within IBM SPSS Statistics 20 programming environment.

**Results:**

The 16 multiple-choice question instrument successfully attained a content validity index of 0.97, showing satisfactory internal consistency, with 0.61 Cronbach's alpha [95% CI 0.462-0.746] and an intraclass correlation coefficient of 0.869 [95% CI: 0.789-0.919] between the test and retest scores.

**Conclusion:**

The CSII – Brazil instrument is considered adequate and validated to assess continuous subcutaneous infusion system users’ conceptual and procedural knowledge.

## INTRODUCTION

Treatment for type 1 diabetes mellitus (T1D) has made significant progress over the past 50 years, mainly with the advent of the continuous subcutaneous insulin infusion (CSII) in the 90's, which allows for the patient to obtain more accurate doses of insulin, comfort and safety (1).

A number of patients with T1D can be candidates for CSII use, but for successful treatment, users need to be the target of continuous training and monitoring regarding proper use of the system (2).

CSII users must be instructed about basic concepts related to terms appearing in the device menu and messages prompted by patient's use (conceptual knowledge) as well as essential actions to be performed to use the device (procedural knowledge), such as to how to insert and connect the infusion system and how to set up basic parameters such as time, date, basal insulin doses, food and glycemic correction boluses. Users are also required to learn how to program advanced settings (such as temporary basal rates, extended/dual/square wave), be capable to identify alarms and solve the most common problems involving CSII use, such as cannula or catheter occlusion and non-administration of insulin. Furthermore, users must also be instructed on how to properly dispose of waste yielded by supplies in proper packaging (3).

CSII is a high-cost and delicate management treatment, but it offers numerous resources for users who manage to learn how to make use of it. Training and education by healthcare providers is thus a constant need in order to avoid the risk of poor glycemic control, diabetic ketoacidosis, lipodystrophy and non-adherence to treatment (4).

An instrument to assess patients’ knowledge and use of CSII is an excellent tool in diabetes education, as it allows a more objective way to evaluate users’ understanding of how the system works and how to use it. In order to ensure instrument quality and reliability, validity must be measured (5). An instrument for this purpose has not been found in the literature.

The aim of this study was to develop, culturally adapt, and validate an instrument to assess users’ conceptual and procedural knowledge of CSII (CSII – Brazil).

## MATERIALS AND METHODS

This is a methodological and exploratory study carried out from July 2017 to August 2019 in the city of Belo Horizonte, State of Minas Gerais, Brazil. The project was approved by the Ethics and Research Committee Involving Human Beings (CAAE number 65656117.6.1001.5138) at Santa Casa of Belo Horizonte Hospital. Agreement to participate in the study was obtained by using a Free Informed Consent Form signed by participants upon accessing an electronic questionnaire within the *e-Surv* webtool.

### Stage 1 – Instrument development

Based on national and international T1D guidelines, in addition to user manuals and instructions provided by different CSII models and brands, a panel of researchers with expertise in diabetes, comprising a dietitian, a nurse and two endocrinologists, developed the instrument and assessed all stages until the final version (6–10).

**Figure 1:**
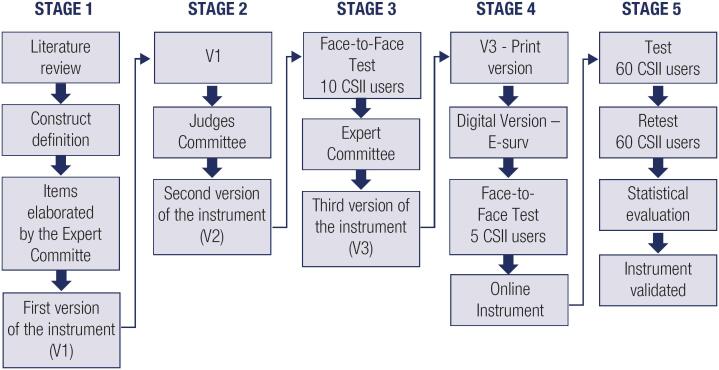
Steps in the process of preparing and validating the instrument CSII.

Specific topics on CSII use were identified for testing in the questionnaire and a first version (V1) was drafted with 17 questions. The instrument was designed to be brief and a self-administered questionnaire (Figure 1).

In establishing a general conceptual structure, the instrument was developed in two parts: the first one made up of yes or no questions aimed to assess self-reported basic conceptual knowledge about T1D concepts which are referred to in the device menu and messages prompted by patient's use. These comprised: sensitivity factor, insulin-carbohydrate ratio, glycemic objective, active insulin time and others. The reason for having this first part was the need to assess whether users report being able to understand basic concepts of their treatment related to the terms used to name them in the device.

The second part comprised questions on user procedural knowledge about CSII therapy, ranging from dealing with basic systems (technique and regularity of the exchange of supplies, adjustment of date and time, administration of basal doses and bolus of insulin, correct disposal of supplies and special bolus programming, temporary basal rates and other advanced CSII settings).

### Stage 2 – Assessment by expert committee

For content validation, a link to access the instrument on the web platform *e-surv* was sent by e-mail to 27 experts (labelled as Judges Committee), among them endocrinologists (n = 10), nurses (n = 3) and dietitians (n = 7) with experience in CSII therapy, as well as applied linguists (n = 7) from different regions of Brazil, who assessed each item in V1 and rated them with the following options: One star standing for need for full reformulation; Two stars, partial reformulation (substantial revision needed); Three stars, need for partial reformulation, with minor editing to enhance text style; and Four stars – no need for reformulation. A comment box was also provided for the experts’ considerations and suggestions.

Once assessment was concluded, the Content Validity Index (CVI – the level of agreement of experts on adequacy of the items) was computed: the number of scores 3 and 4, divided by the total number of scores by all members of the committee. Results greater than or equal to 0.78 being considered acceptable (11).

After reviewing V1, following experts’ suggestions, 2 questions were merged into single one, since they dealt with the same topic (general alarm), and version 2 (V2) was obtained, with 16 questions.

### Stage 3 – Cultural adaptation

Cultural adequacy of V2 was verified through a pre-test (face-to-face) with 10 CSII users of a public diabetes center. Each participant was requested to read the whole instrument; subsequently, the participant was asked to point out whether the items were clear, accurate, and relevant. The participant's feedback was discussed by the panel of researchers responsible for developing the questions, who deemed all comments relevant and reformulated those items that reached less than 80% of agreement (12). The reformulated items were subsequently tested in the same group of participants.

When there was no need for further reformulation, either in the number of questions or in their content, and thus no need for a new assessment by the researchers, version (V3) was obtained, which was considered culturally adequate to be submitted to psychometric validation.

### Stage 4 – Adaptation for web access

At this stage, an electronic version was prepared on the web platform *e-Surv*. Five CSII users recruited at a public diabetes center received a link to access and answer a version of the instrument on the Web, to test its use and describe their experience. No technical difficulty in accessing and answering the questionnaire via the *e-Surv* platform was reported.

### Stage 5 – Validation

A link to access an electronic version of the instrument on the *e-Surv* platform was sent via e-mail to 103 patients referred by endocrinologists from public and private diabetes centers from Belo Horizonte, Minas Gerais, Brazil. Exclusion criteria were patients who were under 18 years old and patients who did not answer or answered incorrectly the questions in the first part of the instrument. A retest was performed in the final sample of 60 patients (after exclusions), with a minimum interval of 7 days and a maximum of 21 days between the tests (13).

To assess the number of correct answers, a score of one to three was assigned to each alternative in each question. The coding of participant responses was based on an increasing order, with the lowest score being assigned to the wrong answer and the highest one to the correct answer. The instrument has twelve three-alternative multiple choice questions and four two-alternative multiple choice questions. The maximum expected score if respondent answers all questions correctly is 44 whereas incorrect answers in all questions yields a minimum 16.

### Statistical analysis

Absolute and relative frequencies were used to describe the sample characteristics and the proportion of correct answers. Internal consistency and reproducibility were verified to analyze the reliability of the construct. Cronbach's alpha (CA) was used to assess the internal consistency of the instrument.

The instrument reproducibility was evaluated through test-retest (temporal stability), computing the intraclass correlation coefficient (ICC) and Kappa index were added to the percentage of concordant responses in the test and retest, defined as the ratio between the number of individuals who selected the same answer (regardless of being correct or incorrect) at both test and retest and the total number of individuals (11,12). Floor and ceiling effects were measured by the number of respondents receiving the minimum and maximum scores, respectively.

The significance level adopted for the statistical tests was 5%. For data analysis, SPSS version 20.0 was used.

## RESULTS

### Instrument development

This stage spanned 6 months and consisted in bibliographic search and review and meetings by a panel of researchers. A first version of the instrument made up of 25 questions was drafted, which were eventually reduced, as 7 questions were discarded and 2 of them merged into a single one, for the sake of clarity and convenience. V1 was finalized with 17 questions, which became 16 in V2 and V3, with a number of alternatives per question ranging from 2 to 3 (totaling 44 items – each alternative being considered an item, for the purposes of statistical analysis).

All steps described in the literature were followed in the validation process.

### Assessment by expert committee

Of the 28 members in the expert committee, 35.7% were physicians, 25% dietitians, 25% applied linguists, and 10.7% nurses. Among healthcare professionals, most (71.4%) had treated patients who had used or were using CSII (Table 1).

**Table 1:** Expert committee demographic data

Variables		n (%) n = 27
Gender		
	Female	18 (64.3)
	Male	9 (32.1)
Expertise domain		
	Medicine	10 (35.7)
	Applied linguistics	7 (25.0)
	Nutrition	7 (25.0)
	Nursing	3 (10.7)
First degree obtained		
	Less than 5 years ago	3 (10.7)
	5 to 10 years ago	11 (39.3)
	10 to 20 years ago	11 (39.3)
	Over 20 years ago	2 (7.1)
Graduate education level		
	First degree	2 (7.1)
	Diploma course	7 (25.0)
	Master's degree in progress	3 (10.7)
	Master's degree	7 (25.0)
	Doctoral degree in progress	4 (14.3)
	Doctoral degree	4 (14.3)
Professional practice area		
	Ambulatory and outpatient care	19 (67.8)
	Scientific research	6 (21.4)
	Consulting	2 (7.1)
Prior participation in expert committees		
	No	17 (60.7)
	Yes	10 (35.7)
	Did not answer	1 (3.6)

The instrument achieved good scores by the committee regarding clarity and relevance, with a total CVI of 0.97. For item relevance, the average CVI was 0.99. For clarity, the index was 0.95 (Table 2).

**Table 2:** Total CVI per instrument's item of CSII – Brazil

Description	Relevance	Clarity
How do you manage to deal with CSII time and date settings?	1.00	0.93
How do you manage to deal with pump alarms or alert sounds?	0.96	0.82
In case of an occlusion alarm or non-delivery of insulin, what do you do?	0.96	0.86
How often do you perform hand hygiene with soap and water and/or 70% alcohol when changing disposables?	1.00	0.93
How often do you clean your skin before applying the cannula?	1.00	0.96
How many times do you move the plunger to lubricate the reservoir/cartridge before aspirating insulin?	1.00	0.96
Do you check for and remove air bubbles in the infusion set when changing disposables?	1.00	1.00
Do you remove air bubbles before and after connecting the infusion set to the insulin reservoir?	1.00	1.00
How often do you change your cannula?	1.00	1.00
Do you rotate pump infusion sites?	1.00	0.96
Have you ever had lipodystrophy (nodules, local hardening) at cannula application sites since you started using your pump?	1.00	1.00
Can you set basal insulin doses on your own?	1.00	1.00
Can you activate and program the temporary basal function?	1.00	1.00
Can you set boluses (insulin/carbohydrate ratio, sensitivity factor and glycemic goal) on your own?	1.00	1.00
Can you program bolus types (“dual wave” and “square wave”) on your own?	1.00	1.00
Where do you dispose of supplies (needles, lancets, cannulas, infusion sets, insulin vials)?	0.96	0.89
In case of an emergency do you have spare syringes or application pens, fast and slow insulin, batteries, and disposables readily available for use?	0.96	0.89
CVI (mean)	0.991	0.951
TOTAL CVI PER ITEM	0.99	0.95
TOTAL INSTRUMENT CVI	0.97	

CSII: continuous subcutaneous infusion systems; CVI: Content Validity Index.

### Validation

Among the 60 patients who answered the final version of the instrument (test and retest), age ranged from 18 to 82 years old (36.2 ± 12.43 years) and gender was predominantly female. All patients in the sample had attended elementary school and most of them reported a monthly income of over five times the minimum wage in Brazil. 21,6% of them had purchased their CSII themselves (Table 3). Due to its homogeneity, the instrument was considered as unidimensional. In this context, the total CA alpha value was 0.61 (95% CI: 0.462-0.746). Floor effects (percent with minimum score) were 0%, and ceiling effects (percent with maximum score) 7%.

**Table 3:** Demographic data of participants in psychometric validation of CSII – Brazil

Variables		n (%) n=60
Age (years)		36.2 ± 12.43
Time since diagnosis (years)		19.5 ± 9.3
Time using CSII (years)		6.1 ± 4.7
Gender		
	Female	44 (73.3) 16 (26.6)
	Male	
Education level		
	Unfinished high school	2 (3.3)
	Finished high school	11 (18.3)
	College (degree not awarded)	9 (15)
	College (degree awarded)	38 (63.3)
Monthly family income		
	Up to 1 minimum wage	2 (3.3)
	1 to 2 minimum wages	15 (25)
	3 to 4 minimum wages	14 (23.3)
	Over 5 minimum wages	29 (48.3)
Patient access to CSII		
	Public Healthcare System funded (as result of patient-filed lawsuit)	47 (78.3)
	Privately purchased	13 (21.6)
Medical follow-up		
	Public Healthcare System	14 (23.3)
	Private healthcare insurance system	46 (76.6)
CSII model		
	Medtronic – Paradigm VEO	25 (41.6)
	Medtronic – Minimed	15 (25)
	Roche Accu-Check Spirit combo	12 (20)
	Medtronic – Paradigm 722	5 (8.3)
	Medtronic – Paradigm 715	2 (3.3)
	Roche Accu-Chek Spirit	1 (1.6)

CSII: continuous subcutaneous insulin infusion.

An ICC value of 0.869 (95% CI: 0.789-0.919) was obtained. The Kappa coefficient, which assesses the degree of agreement, varied between 0.5-1.0 (mean: 0.80). When the alpha absence index was calculated, there was a slight impact on reducing AC and no questions were removed (Table 4). The instrument is available in Supplementary Material.

**Table 4:** Correlation between test and retest answers agreement percentage and Cronbach's alpha coefficient for CSII – Brazil

Question	Cronbach's Alpha if item is removed	95% CI for Cronbach's alpha	Percentual agreement test-retest	Kappa index
1	0.601	0.437-0.735	95	0.95
2	0.602	0.438-0.736	96	0.784
3	0.604	0.440-0.736	86	0.711
4	0.55	0.364-0.701	90	0.75
5	0.61	0.452-0.742	80	0.504
6	0.589	0.419-0.726	96	0.861
7	0.622	0.465-0.748	98	0.98
8	0.576	0.401-0.718	93	0.859
9	0.611	0.450-0.741	98	0.793
10	0.628	0.474-0.752	100	1
11	0.644	0.497-0.763	88	0.708
12	0.605	0.442-0.737	91	0.742
13	0.608	0.447-0.739	90	0.669
14	0.593	0.425-0.729	90	0.806
15	0.6	0.435-0.734	83	0.743
16	0.59	0.421-0.727	100	1
TOTAL	0.61	0.462-0.746	92.1	0.803

The average final score during test was 39.4 (31-44 marks). 34 participants (56.6%) scored over 40 (90% correct answers).

Among the 16 questions in the instrument, four of them had a success rate greater than 90%. They deal with the topics: adjusting time and date of the pump whenever necessary, observing information prompted by alarms, eliminating air bubbles in the infusion set when changing disposables, and rotating infusion sites.

Four questions showed less than 70% correct answers. The topics dealt with in them were: measures in case of occlusion alarm (63.3% answered that they performed the change of the entire infusion set); changing the infusion set (63.3% answered that they changed the set every 3 days); special bolus programming (58.3% of patients showed knowing how to program) and disposal location for the infusion set, syringes and needles (only 33.3% of patients performed correct disposal in a rigid plastic container or in a suitable packaging for discard).

## DISCUSSION

CSII is an expensive therapy, not very accessible in Brazil, but a promising one with numerous resources for T1D management, which merits research towards its dissemination. It represents a treatment option that must be carefully prescribed in order to guarantee successful results.

Implementing an instrument with psychometric qualities tested for the clinical practice of diabetes educators will enable a more efficient direction in patient follow-up. In addition to contributing to education of CSII users in the Brazilian cultural context, the design and development of such an instrument can be of fundamental importance in the identification of flaws in relation to the practices oriented to the use of CSII. This evaluation, in turn, will make it possible to readjust the teaching-learning process, so that the use of CSII can be optimized, guaranteeing the benefits that this system can bring to the user.

The collaborative work by an interdisciplinary team (a panel of researchers with expertise in diabetes and CSII) to develop the instrument made it possible to clarify and solve problems encountered during the process of drafting the items, in addition to allowing the adaptation of concepts and terms to the language used by the target subjects.

Assessment by an expert committee through the web tool *e-Surv* proved a reliable and efficient methodology (14). This furthered collaboration between healthcare and applied linguistics experts towards adaptation of the instrument, in addition to offering experts a channel to provide suggestions.

The instrument scored successfully in terms of agreement within the expert committee regarding clarity and relevance, with an excellent CVI (0.97). It should be noted that the maximum value for CVI is equal to 1, so the results obtained are close to the maximum evaluation limit (11).

A face-to-face test with a small group of people (15) proved successful. In carefully developed instruments, two or three face-to-face tests are usually sufficient, which was confirmed in our sample, which required two rounds of tests (16).

As regards internal consistency, a CA index of 0.61 was obtained. A CA index above 0.5 is acceptable when the sample is between 25 to 50 participants; in a basic investigation or exploratory research, a value of around 0.60 would suffice to meet acceptability (17–19). Several studies report interferences to which the CA index is subject, which demands caution in its interpretation. It is worth noticing that instruments with a large number of items or samples have higher CA values (20–22).

The authors believe that an adequate CA index was achieved, considering the context in which the study was performed, with a restricted sample due to the fact that the therapeutics is still poorly available in our country (12).

The CA absence index was carried out, but no questions were excluded, due to the small difference that would result in the final CA. As ours is a brief instrument, with a reduced number of questions pertaining to a single domain, removing a question would have a negative impact; thus, all of them were considered essential by the panel of experts.

The results showed good stability of the instrument, with a high level of agreement between test and retest (all questions showed more than 80% agreement). The ICC (0.869) and the Kappa index (mean 0.80) proved that the instrument showed stability, reproducibility and confidence (11).

No studies were found describing the development and validation of instruments to assess the knowledge of CSII users, which did not allow for our results to be compared.

The first part of our instrument seeks to elicit users’ self-reported conceptual knowledge deemed necessary for a successful treatment with insulin. In the case of CSII, it is important that users be familiar with terms that the system displays as messages. Assessing clear understanding of these terms is then essential to adequate use of the system.

For the validation stage, patients who scored poorly in part 1 were excluded for retest. Daily clinical practice recommends that, whenever a patient is in doubt or does not know how to answer a question, administration of instruments must be interrupted so that the patient's doubt is clarified, and the instrument can be newly applied after clarification. This is seen as a moment to improve or revise concepts that may have not be consolidated.

The second part of the instrument was meant to assess CSII users’ procedural knowledge regarding decisions they are required to make when using a CSII.

The instrument is intended to be self-administered and in patient familiar settings, such as a physician's waiting room. Feedback provided by the instrument allows endocrinologists to identify the difficulties faced by a patient and, even during clinical consultation.

When we analyzed the performance of the patients in the test, the question that obtained the lowest rate of correct answers did not surprise us; this was related to proper handling or correct disposal of sharps. A solution to incorrect behavior by T1D patients is to instruct and train them (23,24).

It was also expected that the question about the user's ability to deal with special boluses (double/square, wave/prolonged) would have the lowest hit rates, as this is not frequently addressed in patient education or training.

Currently in Brazil we are experiencing constant delays in the delivery of supplies, and while developing this instrument we have noticed how patients make wrong decisions due to this, especially regarding inappropriate use of catheters and cannulas for longer than recommended. The risk of complications such as infections and interruption of insulin administration increases with this poor practice.

As regards other items in the instrument, participants’ performance was homogeneous and with good success rates; however, there are some gaps in the skills of these patients, which points to the need to intensify care and education.

In conclusion, the instrument proved useful, reliable and stable for application to users with CSII to assess conceptual and procedural knowledge with a view to enhancing health care practices.

## Supplementary Material

INSTRUMENTO SICI – BRASIL

Qual a marca e o modelo da bomba de Insulina que você utiliza? Marca______________________ Modelo______________________

**ETAPA 1:** Você sabe o significado dos termos abaixo?

Dose total diária de insulina	Sim	( )	Não	( )
Dose total diária de insulina basal	Sim	( )	Não	( )
Relação insulina/carboidrato	Sim	( )	Não	( )
Meta glicêmica	Sim	( )	Não	( )
Fator de sensibilidade	Sim	( )	Não	( )
Tempo de insulina ativa	Sim	( )	Não	( )

Caso alguma resposta da questão anterior tenha sido NÃO, pare o preenchimento. Informe ao profissional de saúde, para esclarecimento da dúvida, antes de continuar a responder.

**ETAPA 2:**

01	Em relação ao ajuste da hora e data da bomba, marque a resposta com a qual você se identifica:
○	Não me preocupo com hora e a data da bomba.
○	Ajusto a hora e a data da bomba sempre que tiver necessidade, por exemplo, no horário de verão e em outros fusos horários.
○	Não sei como ajustar a hora e a data da bomba.
	
02	Quando qualquer alarme da bomba dispara, o que você faz?
○	Silencio o alarme e sempre observo a informação fornecida pela bomba.
○	Silencio o alarme e nem sempre observo a informação fornecida pela bomba.
○	Não sei o que fazer.
	
03	Em caso de alarme de oclusão ou de não administração de insulina, o que você faz?
○	Troco apenas a cânula e/ou cateter
○	Troco a cânula, cateter e reservatório
○	Não sei o que fazer e entro em contato com a equipe de saúde
	
04	Você faz a higiene das mãos antes de trocar os descartáveis?
○	Todas as vezes
○	De vez em quando
○	Nunca
	
05	Você faz a higiene da pele antes de aplicar a cânula?
○	Todas as vezes
○	De vez em quando
○	Nunca
	
06	Antes de aspirar a insulina, quantas vezes você movimenta o êmbolo para lubrificar o reservatório/cartucho?
○	Nenhuma
○	1-2 vezes
○	3 vezes ou mais
	
07	Durante a troca de descartáveis, você observa e elimina as bolhas de ar do conjunto de infusão?
○	Sim
○	Não
○	Às vezes
	
08	Você faz a troca da cânula com qual frequência?
○	De 2 em 2 dias
○	De 3 em 3 dias
○	De 4 em 4 dias ou mais
	
09	Você faz rodízio dos locais de aplicação da cânula?
○	Sim
○	Não
○	Às vezes
	
10	Após o início do uso da bomba de insulina, você percebeu endurecimento ou qualquer alteração nos locais de aplicação da cânula?
○	Sim
○	Não
	
11	Você precisa de ajuda para configurar as doses de insulina basal na bomba?
○	Sim
○	Não
	
12	Você sabe ativar e programar a função de dose basal temporária?
○	Sim
○	Não
○	Não sei o que é isso
	
13	Você sabe configurar sem ajuda os bolus (relação insulina/ carboidrato, fator de sensibilidade, meta glicêmica e tempo de insulina ativa)?
○	Sim
○	Não
	
14	Você sabe programar sem ajuda os bolus especiais (duplo/ multionda e quadrado/ prolongado)?
○	Sim
○	Não
○	Não sei o que é isso
	
15	Onde você descarta as agulhas, lancetas, cânulas, conjuntos de infusão e frascos de insulina?
○	Todos em lixo comum.
○	Todos em recipiente plástico rígido ou embalagem própria para descarte.
○	Apenas materiais cortantes em recipiente rígido ou embalagem própria para descarte, restante em lixo comum.
	
16	Em caso de necessidade, você tem sempre disponível o kit de emergência (seringas ou canetas de aplicação, insulina rápida e lenta, pilhas e materiais para a troca de descartáveis)?
○	Sim
○	Não

**Table:** CODIFICAÇÃO

01	Em relação ao ajuste da hora e data da bomba, marque a resposta om a qual você se identifica:
2	Não me preocupo com hora e a data da bomba.
3	Ajusto a hora e a data da bomba sempre que tiver necessidade, por exemplo, no horário de verão e em outros fusos horários.
1	Não sei como ajustar a hora e a data da bomba.
	
02	Quando qualquer alarme da bomba dispara, o que você faz?
3	Silencio o alarme e sempre observo a informação fornecida pela bomba.
2	Silencio o alarme e nem sempre observo a informação fornecida pela bomba.
1	Não sei o que fazer.
	
03	Em caso de alarme de oclusão ou de não administração de insulina, o que você faz?
2	Troco apenas a cânula e/ou cateter
3	Troco a cânula, cateter e reservatório
1	Não sei o que fazer e entro em contato com a equipe de saúde
	
04	Você faz a higiene das mãos antes de trocar os descartáveis?
3	Todas as vezes
2	De vez em quando
1	Nunca
	
05	Você faz a higiene da pele antes de aplicar a cânula?
3	Todas as vezes
2	De vez em quando
1	Nunca
	
06	Antes de aspirar a insulina, quantas vezes você movimenta o êmbolo para lubrificar o reservatório/cartucho?
1	Nenhuma
2	1-2 vezes
3	3 vezes ou mais
	
07	Durante a troca de descartáveis, você observa e elimina as bolhas de ar do conjunto de infusão?
3	Sim
1	Não
2	Às vezes
	
08	Você faz a troca da cânula com qual frequência?
2	De 2 em 2 dias
3	De 3 em 3 dias
1	De 4 em 4 dias ou mais
	
09	Você faz rodízio dos locais de aplicação da cânula?
3	Sim
1	Não
2	Às vezes
	
10	Após o início do uso da bomba de insulina, você percebeu endurecimento ou qualquer alteração nos locais de aplicação da cânula?
1	Sim
2	Não
11	Você precisa de ajuda para configurar as doses de insulina basal na bomba?
1	Sim
2	Não
	
12	Você sabe ativar e programar a função de dose basal temporária?
3	Sim
2	Não
1	Não sei o que é isso
	
13	Você sabe configurar sem ajuda os bolus (relação insulina/carboidrato, fator de sensibilidade, meta glicêmica e tempo de insulina ativa)?
2	Sim
1	Não
	
14	Você sabe programar sem ajuda os bolus especiais (duplo multionda e quadrado/ prolongado)?
3	Sim
2	Não
1	Não sei o que é isso
	
15	Onde você descarta as agulhas, lancetas, cânulas, conjuntos de infusão e frascos de insulina?
1	Todos em lixo comum.
3	Todos em recipiente plástico rígido ou embalagem própria para descarte.
2	Apenas materiais cortantes em recipiente rígido ou embalagem própria para descarte, restante em lixo comum.
	
16	Em caso de necessidade, você tem sempre disponível o kit de emergência (seringas ou canetas de aplicação, insulina rápida e lenta, pilhas e materiais para a troca de descartáveis)?
2	Sim
1	Não
